# Supplementary figures and images for: Identifying New Contributors to Brain Metastasis in Lung Adenocarcinoma: A Transcriptomic Meta-Analysis
Source: Cancers (Basel). 2023 Sep 12;15(18):4526. doi: 10.3390/cancers15184526 (PMC10526208; doi:10.3390/cancers15184526)

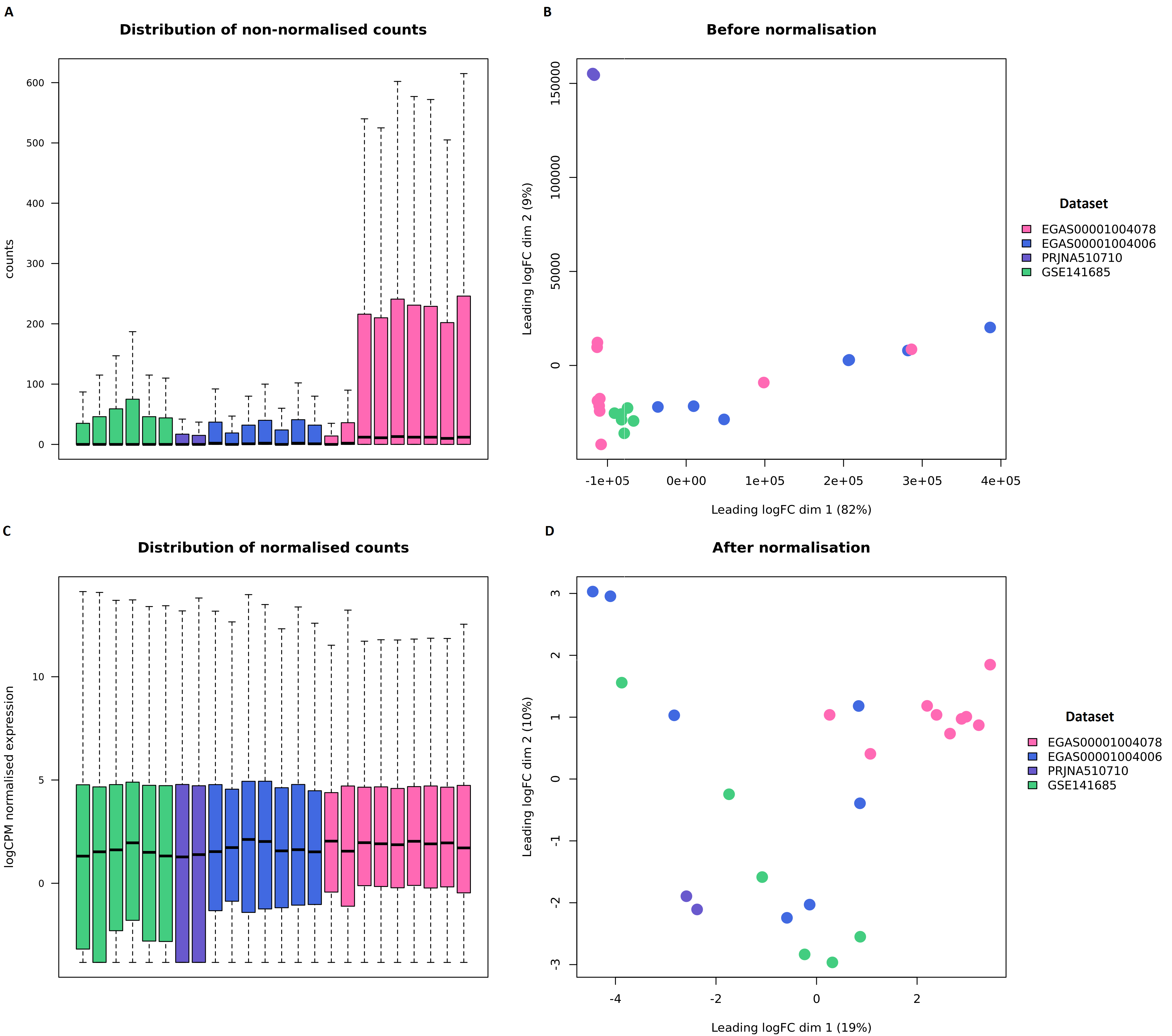

Supplement: Supplementary file 1 [file cancers-15-04526-s001.zip › Supplementary information/Figure S1.png]

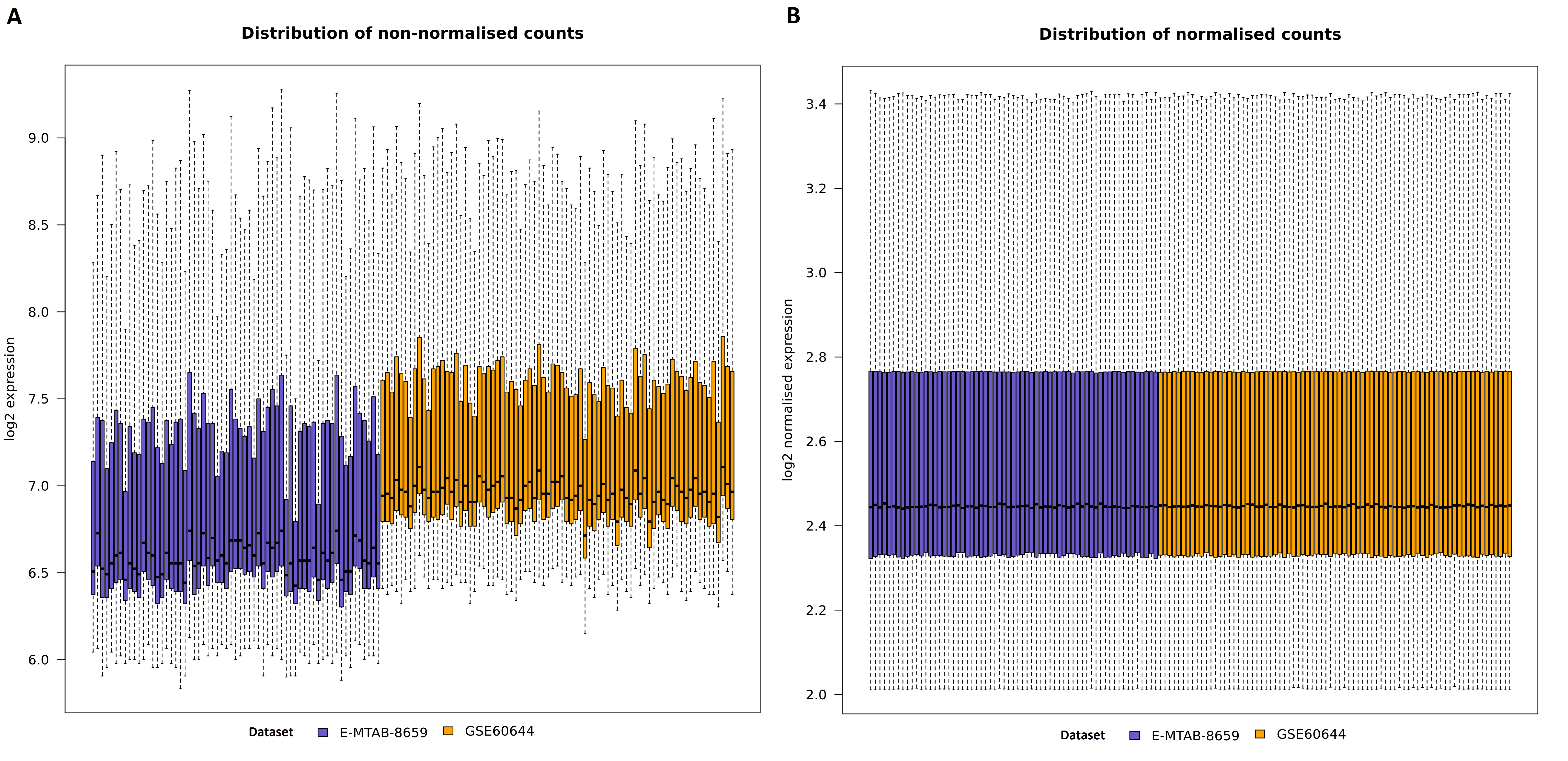

Supplement: Supplementary file 1 [file cancers-15-04526-s001.zip › Supplementary information/Figure S2.png]

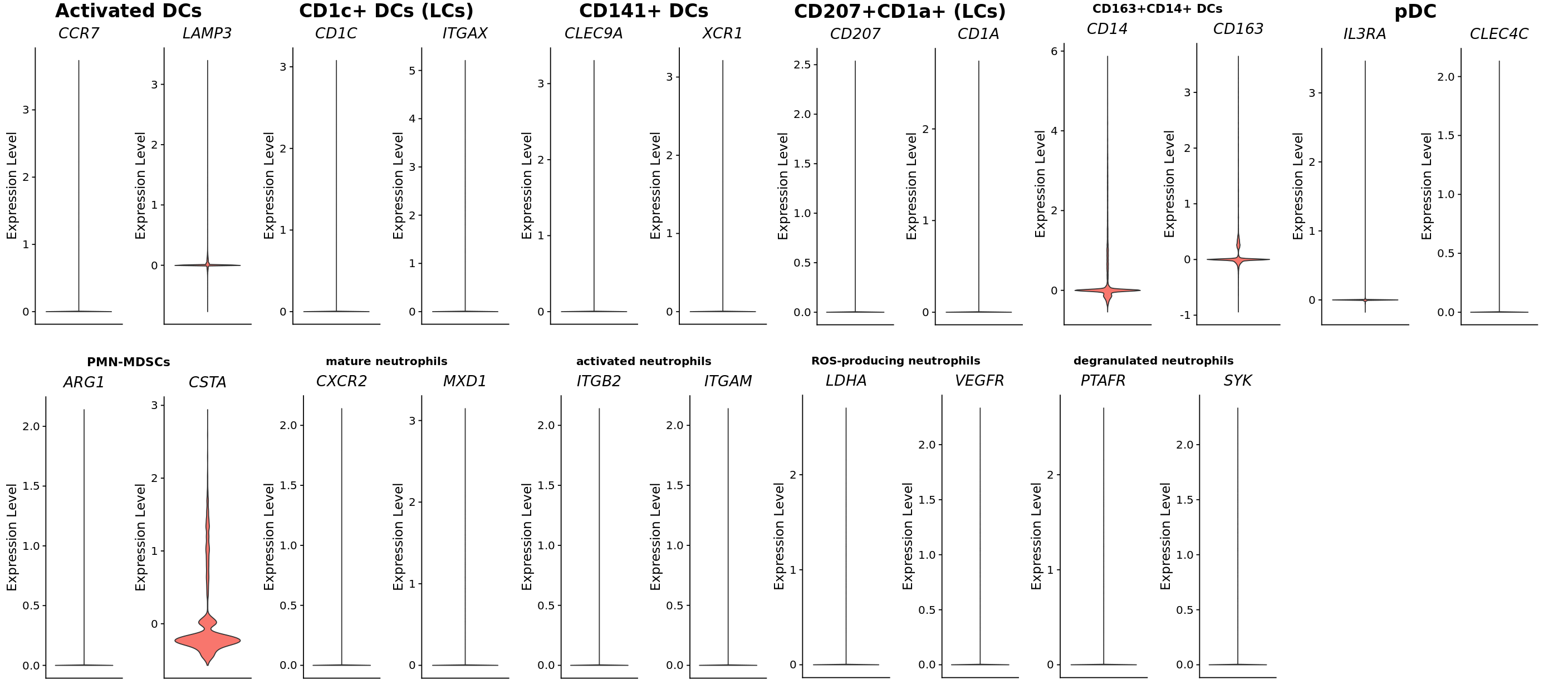

Supplement: Supplementary file 1 [file cancers-15-04526-s001.zip › Supplementary information/Figure S5.png]

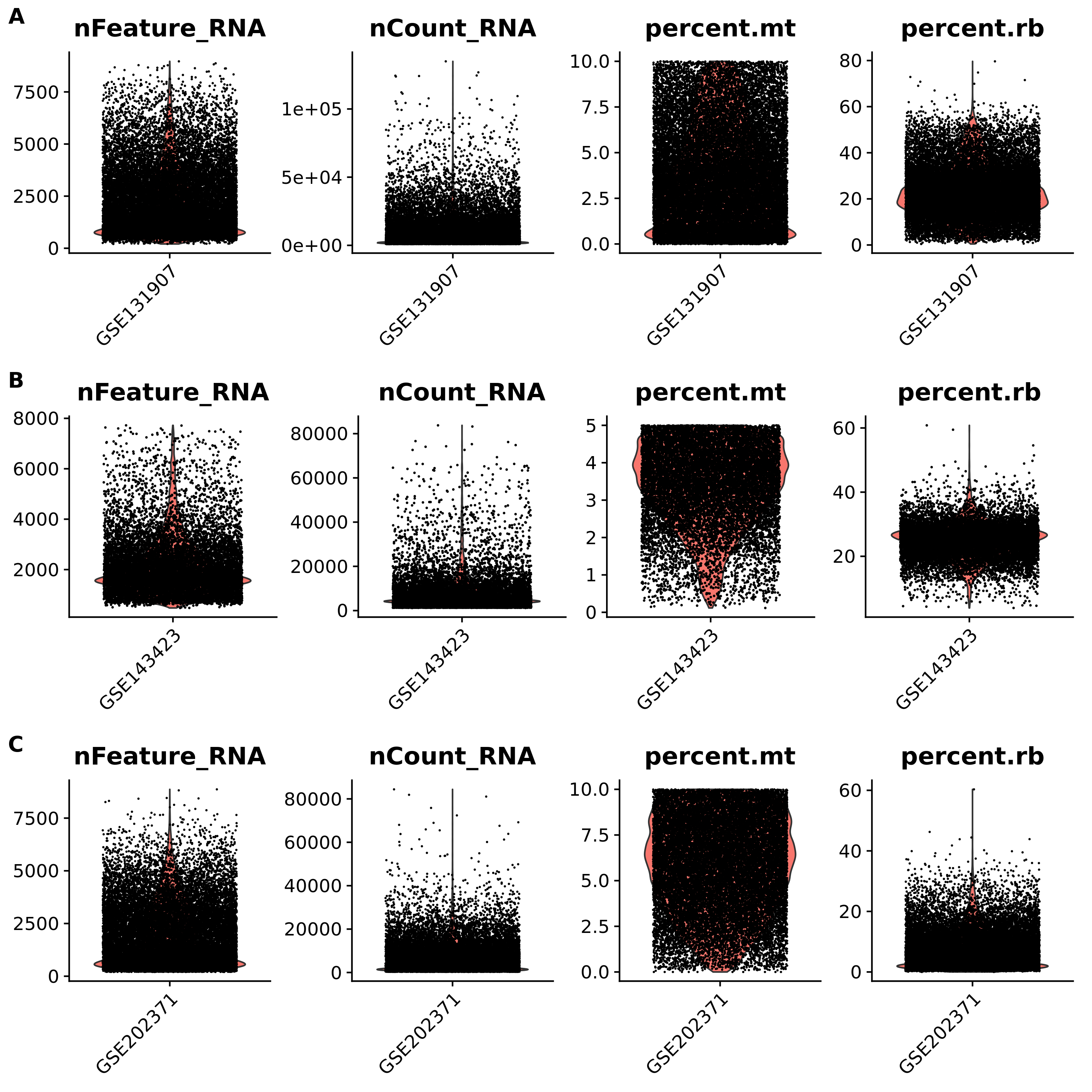

Supplement: Supplementary file 1 [file cancers-15-04526-s001.zip › Supplementary information/Figure S3.png]
